# Larvicidal, Histopathological, Antibacterial Activity of Indigenous Fungus *Penicillium* sp. Against *Aedes aegypti* L and *Culex quinquefasciatus* (Say) (Diptera: Culicidae) and Its Acetylcholinesterase Inhibition and Toxicity Assessment of Zebrafish (*Danio rerio*)

**DOI:** 10.3389/fmicb.2019.00427

**Published:** 2019-03-18

**Authors:** Chinnasamy Ragavendran, Venkatesan Manigandan, Chinnaperumal Kamaraj, Govindasamy Balasubramani, Joy Sebastian Prakash, Pachiappan Perumal, Devarajan Natarajan

**Affiliations:** ^1^Natural Drug Research Laboratory, Department of Biotechnology, School of Biosciences, Periyar University, Salem, India; ^2^Biomedical Zebrafish Laboratory, Department of Medical Biotechnology, Faculty of Allied Health Sciences, Chettinad Academy of Research and Education, Chennai, India; ^3^Marine Biotechnology and Ecological Genomics Laboratory, Department of Biotechnology, School of Biosciences, Periyar University, Salem, India

**Keywords:** larvicidal, acetylcholinesterase, histopathology, antibacterial, zebrafish embryo, FTIR, GCMS

## Abstract

Fungal metabolites are considered to be most efficient tools to overcome the issues related to insecticide resistance and environmental pollution. The present study focus on the evaluation of the mosquito larvicidal efficacy of metabolites of seven indigenous fungal isolates (*Penicillium* sp. *Aspergillus niger*, *A. flavus*, *A. parasiticus*, *Rhizopus* sp. *Mucor* sp. and *Aspergillus* sp.) on the larvae of *Aedes aegypti* and *Culex quinquefasciatus* under the laboratory condition. The preliminary screening of the isolate, *Penicillium* sp. showed better larvicidal effect when compared to other fungi. The fungus was grown on Potato Dextrose Broth (PDB) in the laboratory (at 25°C) and maintained in the relative humidity (at 76 ± 4% for 15 days). Larvicidal potency of mycelial ethyl acetate extract (MEAE) of *Penicillium* sp. was performed against 1st to 4th instars larvae of *Ae. aegypti* and *Cx. quinquefasciatus* using four different concentrations (100, 200, 300, and 500 μg/ml) that showed better larval mortality values (μg/ml) of LC_50_ = 6.554, 5.487, 6.874, 6.892, and the LC_90_ = 11.486, 10.366, 12.879, 13.865 for *Ae. aegypti* and LC_50_ = 7.000, 13.943, 18.129, 25.212 and the LC_90_ = 12.541, 23.761, 30.923, 41.696 for *Cx. quinquefasciatus*. Exposure of metabolite to larvae resulted in behavior changes i.e., excitation, up and down with aggressive movement, anal papillae biting behavior. Further, the larvae treated with *Penicillium* sp. metabolite exhibited significant reduction in the levels of acetylcholinesterase. The 4th instar mosquito larvae treated with the 500 μg/ml mycelia extract showed severe histological damages. During the antibacterial analysis of *Penicillium* sp.- mycelium the maximum growth inhibition zone was recorded in *Shigella dysenteriae* (31.2 mm) and *Klebsiella pneumoniae* (31.1 mm) followed by others. In addition, to check the toxicity of *Penicillium* sp. MEAE against embryos of Zebrafish, a model system, using different concentrations of metabolites (1.0, 0.5, 0.125 mg/ml, 30, 3.0, and 0.5 μg/ml) and life-stage parameters were observed at 124 hpf. Furthermore, the Fourier Transformed Infrared and GCMS spectrum analysis of mycelium reflected several chemical compounds. The outcome of the study clearly shows that *Penicillium* sp. metabolites could serve as an ideal eco-friendly, single-step and inexpensive source for the control of *Ae. aegypti* and *Cx. quinquefasciatus* larvae.

## Introduction

Globally, mosquitoes are one of most life threatening insects, as they are major vectors for the cause of several diseases (Ghosh et al., 2013; Benelli et al., 2016; Benelli and Romano, 2017; Ward and Benelli, 2017). The dengue vector, *Aedes aegypti*, distributed in tropical and sub-tropical regions of the world has the potential to transmit several viruses that cause Zika, dengue, chickungunya and yellow fever (Reegan et al., 2015; Benelli et al., 2017a,b). Another mosquito, *Culex quinquefasciatus* (Say) belonging to the family, Culicidae is predominantly found in the tropical and temperate regions (Bernhard et al., 2003). This mosquito could transmit the Lymphatic filariasis to humans and which also plays an important role in the transmission of West Nile Virus (Tsai and Mitchell, 1989; Reuben et al., 1994).

Approximately, 390 million people are now at risk of the dengue fever (WHO, 2011). Another (Brady et al., 2012) study proved the prevalence of dengue and they estimate that 3.9 billion peoples in 128 countries are at risk of infection with dengue virus. *Wuchereria bancrofti*, the lymphatic dwelling parasite infected about 90 million people worldwide (Kovendan et al., 2009). 40% of the filarial cases occur in India which results in an annual economic loss of about 720 crores (Hotez et al., 2004).

For the management of mosquitoes and houseflies we mainly depend on the application of synthetic and commercial chemicals viz; organochlorines, organophosphates, carbamates, Dichloro Diphenyl Trichloroethane’s (DDT) and others, especially to control their increased resistance and to arrest the severe side effects (Wood, 2003; Harzsch and Hafner, 2006; Benelli, 2015a,b; Naqqash et al., 2016). Moreover, the cost of synthetic pyrethroids are very high and they cause environmental and food safety issues besides they affect non-target organisms (Mittal and Subbarao, 2003; Pates and Curtis, 2005; Nathan et al., 2006; Benelli, 2015b). More intensive researches have been recently carried out to control mosquito vectors in an holistic way (Singh et al., 2006). Hence, the scientists from across the world, have been searching for an alternative method for vector control (Vyas et al., 2007). The utilization of natural products or metabolites from microbial resources is a possible alternative way, which is environmentally safe, biodegradable, low cost larvicidal agent for vector control (Killeen et al., 2002; Scholte et al., 2005). Fungal based products have been reported as highly toxic to mosquitoes, with less side effects to non-target organisms (Kirschbaum, 1985). Generally, microbial insecticides are considered as alternative to chemical insecticides, because of their selective toxicity, decomposability, and eco-friendly nature (Misato, 1983; Shaalan et al., 2005). Mycelial extract of various fungi have been reported for their larvicidal, cellulolytic and cytotoxic activity (Valentin Bhimba et al., 2011; Zhang et al., 2013; Fahd, 2018). The most commonly known fungal species which possess significant larvicidal activity are; *Penicillium* sp. *Aspergillus* sp. *Fusarium* sp. *Podospora* sp. *Mucor* sp. *Cladosporium* sp. and *Stoloniferum* sp. (Dennis et al., 2013).

The increasing prevalence of antibacterial resistance among microbes against commercial antibiotics has become a major health issue globally. Although various new antibiotics were developed during recent decades but none of them found effective on multidrug-resistant bacteria (Mohanty et al., 2012). Therefore, an intensive search is needed to treat Gram-positive and negative human pathogens. Fungi are being considered as best sources of bioactive metabolites (Schulz et al., 2002; Strobel, 2003) as they could possess significant antimicrobial agents (Chomcheon et al., 2005; Li et al., 2005; Phongpaichit et al., 2007; Weber et al., 2007).

The zebrafish (*Danio rerio*) is a well-established vertebrate model organism for testing the toxicity of metabolites or nanoparticles toxicant (Deng et al., 2009). The characteristic features like maximum fecundity, controlled and visible embryological phase or internal organs and reactive to toxicants have been the advantages of zebrafish embryos as to use it as model organism in the field of toxicology and pharmacology (Hallare et al., 2006; Chandrasekar et al., 2011; López-Romero et al., 2012; Xu et al., 2014; Yang et al., 2014; Schmidt et al., 2015). Interestingly, the genetic composition of zebra fish is similar to human and approximately 84% of gene associated with human diseases (Howe et al., 2013; Truong et al., 2014). In view of the above, the present study was designed to isolate and identify the potent indigenous soil fungi to evaluate their metabolites larvicidal and histopathological effects on mosquitoes.

## Materials and Methods

### Chemicals and Reagents

The necessary chemical/reagents like Potato Dextrose Agar (PDA), PDB, Muller Hinton Agar (MHA), Nutrient broth (NB), Lactophenol cotton blue stain, acetylthiocholine iodide (AChEI), fast blue B, Sodium dodecyl sulfate (SDS), 5,5-dithiobis-2-nitrobenzoic acid (DTNB), and 10% Dimethyl sulfoxide (DMSO) were purchased from Hi-Media, Mumbai, India. The deionized water was used throughout the bioassay. All the glasswares were washed with diluted nitric acid (HNO_3_), followed by distilled water and dried in hot air oven.

### Collection of Soil Sample

The soil samples were collected from the Karumandurai hills, Salem District Tamil Nadu, India (Latitude 78°20′E and Longitude 11°45′N). Soil samples (approximately 100 g) were aseptically collected (about 5–7 cm depth) using sterile polyethylene bags and brought to the laboratory. Then, it was stored in refrigerator at 4°C until further use.

### Isolation of Fungi by Serial Dilution Method

Fungal isolation was done on PDA medium enriched with antibiotic (Chloramphenicol 1 mg/ml) to arrest the bacterial growth. The culture medium was poured on petri plates and allowed to solidify (Apinis, 1963). Serial dilution of the soil sample (up to 10^−6^ dilution) was carried out using sterile distilled water. To enhance the fungal isolation, sterile pipette was used, 30% of dilution was taken and introduced into the surface of the agar medium and uniformly spread out using sterile L-rod. All the PDA plates were incubated (for 3 days) at room temperature (27 ± 1.5°C) for fungal growth. The growth of various fungal colonies were observed on each plate and they were then isolated separately by the modified plating method of Durowade et al. (2007).

### Point Inoculation Method

The isolated fungi were purified by adopting point inoculating method on plates containing PDA medium and each fungus was purified by repeating the step. Pure form of the isolated fungi was confirmed by microscopic examination of the culture (at 40× magnification) through light microscope. Further, the fungal cultures were sub-cultured on PDA slants incubated for a period of 5–7 days and the cultures were stored at 4°C (Gilman, 1957).

### Macroscopic and Microscopic Characteristics

On the basis of colony morphology and microscopic characteristics, the fungal cultures were stained using a Lacto phenol cotton blue (LCB) stain (Madavasamy and Panneerselvam, 2012). The stained cultures were observed under microscope and the isolated fungi identified using staining method and also checked with available fungal identification manual (Gilman and Abbott, 1957). The following morphological characteristics were taken for identification i.e., colony growth (length and width), occurrence of aerial mycelium, colony color, presence of wrinkles and furrows and pigment production etc. (Abutaha et al., 2015).

### Mycelial Metabolite Preparation

The mycelial mats of the isolated fungi were inoculated into PDB, and incubated for 3 weeks under dark condition. Once the maximal growth was obtained, the fungal mycelial mat was harvested by filtration. The fungal mycelium (10 g) was extracted with 100 ml of ethyl acetate in static condition for 5 days. The mixture was filtered through muslin cloth and the extraction steps were repeated for 3 times. The concentrated mixture was centrifuged at 12,000 rpm for 5 min to remove the unwanted debris. Then, the mixture was transferred to a round bottom flask and dried under rotary evaporator at (40°C), and stored at −20°C until further use (Belofsky et al., 2000; Josphat et al., 2011).

### Collection, Maintenance and Larvicidal Activity of Mosquitoes

The test mosquito larvae were collected from agricultural fields, Karuppur Panchayat, Salem and the larvae were identified as per the mosquitoes key manual (Rueda, 2004; Tyagi et al., 2012). Then, they were maintained in the Natural Drug Research Laboratory (NDRL) under the temperature of 25 ± 2°C, relative humidity at 75 ± 2% and photoperiod of 14:10 (L/D). The mycelial extract was prepared in defined concentrations (100, 200, 300, and 500 μg/ml) and tested for larvicidal activity against targeted mosquitoes (Ragavendran et al., 2017b). Mortality and survival rate was recorded after 24 h of the exposure. During the experimental period, no food was provided to the larvae. All the experiments were performed in thrice to validate results. All test containers were tightly covered with mosquito net and kept at room temperature (avoiding exposure of sunlight) and the dead larvae were counted (WHO, 2005).

### Dose-Response Bioassay

For dose response bioassay, *Ae. aegypti* and *Cx. quinquefasciatus* larvae were taken in beakers containing sterile deionized water. Then, various concentrations of metabolites were prepared using 100 ml water. The mycelial metabolite was dissolved in 10% DMSO for prepared at 1 mg/ml concentration (stock solution 5 ml) and bioassays experiment was performed using different concentrations (100, 200, 300, and 500 μg/ml) of metabolites from mycelium extract (Chandrasekar et al., 2011). The negative control of each experiment (treated with DMSO-distilled water) was tested for three times. The mortality (using Abbott’s formula) and survival rate was determined after 24 h of the exposure (Abbott, 1925). All the test containers were kept at room temperature without any disturbances. The LC_50_ and LC_90_ values of exposed larvae were calculated using probit analysis (Finney, 1971).

$$\mathrm{Corrected mortality}=\frac{\mathrm{Observed mortality}\;(\mathrm{treatment})-\mathrm{Observed mortality}\;(\mathrm{control})}{100-\mathrm{Control mortality}}\times 100$$

$$\mathrm{Percentage mortality}=\frac{\mathrm{Number of dead larvae}}{\mathrm{Number of larvae introduced}}\times 100$$

### Behavioral Studies in Metabolites Against *Ae. aegypti* and *Cx. quinquefasciatus* Larvae

In larvicidal bioassay, the larvae were observed carefully for behavioral changes i.e., wriggling speed, horizontal and vertical movements and self-biting behaviors. The larval behavior symptoms were recorded and photographed with Nikon D-SLR Digital Camera (Nikon Inc., Japan). Similarly, control larvae were also observed and compared with treated larvae (Warikoo and Kumar, 2013).

### Preparation of Whole Body Homogenates

The *Penicillium* sp. extract treated 4th instar larvae were washed with sterile distilled water, and dried with the help of blotting tissue paper. Then, it was homogenized in eppendorf tubes using a mortar and pestle by adding l ml of ice-cold sodium phosphate buffer (20 mM, pH 7.0) for esterases activity. The whole body homogenates were centrifuged (8000 rpm; 4°C) for 15 min, and the clear supernatants were used to perform the enzyme assay.

### Acetylcholinesterase Assay

For this assay, the larval homogenate supernatants were measured by spectrophotometer (at 412 nm) by following the modified method of Ellman et al. (1961) and the acetylthiocholine iodide was served as substrate (Ikezawa and Taguchi, 1981). Acetylcholinesterase activity inhibition was calculated by the formula:

$$\mathrm{Acetylcholinesterase activity inhibition}\;(\%)\;=\;({\text{A}}_{\text{control}}-{\text{A}}_{\text{sample}})/{\text{A}}_{\text{control}}\times 100$$

$${\text{A}}_{\text{control}}\;\mathrm{and}\;{\text{A}}_{\text{sample}}=\;\mathrm{the absorbance of the control sample}\text{.}$$

### Morphology and Histopathology Study

The 4th instar larvae of the test mosquitoes were treated with 500 μg/ml of *Penicillium* sp. mycelia metabolites. The morphological and behavioral changes of the treated larvae were identified using Labomed microscope (at 40×). The dead larvae were mounted on a microscopic slide with Hoyer’s medium and observed under the microscope (Kamalakannan et al., 2014). The control and treated larvae were fixed with 10% formalin, dried out using ethyl alcohol series, cleaned by xylene and fixed by using paraplast and taken section (7 μm). The sectioned larvae and control samples were stained with eosin and hematoxylin, respectively by adopting standard staining protocols (Kaewnang-O et al., 2011; Feldman and Wolfe, 2014). The mid-guts of control and treated larvae were visualized through microscope (Labomed) and photographed.

### Antibacterial Activity Test

#### Collection and Maintenance of Broth Culture

Two Gram-positive (*Staphylococcus aureus* MTCC 737 and *Enterococcus faecalis*) and five Gram-negative (*Escherchia coli* MTCC 443, *Salmonella typhi* MTCC 3224, *Shigella boydii* MTCC 11947, *Shigella dysentriae* and *Klebsiella pneumoniae* MTCC 4030) were procured from the Microbial Type Culture Collection (MTCC), Institute of Microbial Technology, Chandigarh, India. The bacteria cultures were maintained in NA slants (at 4°C) and served as stock cultures. The selected bacteria were inoculated into MHB and incubated at 37°C and 190 rpm for 10–14 h. The turbidity of the resulting suspension was diluted in MHB and matched with 1 McFarland turbidity standard. The resultant level of turbidity was diluted with MHB and is equivalent to approximately 3.0 × 10^−8^ CFU/mL (0.5 MacFarland standards).

#### Agar Well Diffusion Method

The antibacterial activity of MEAE of *Penicillium* sp. was evaluated by agar diffusion method (Thornberry, 1950). Approximately, 25 ml of MHA medium was poured in the sterilized Petri dishes. The selected bacterial cultures were grown in MHB for 24 h. The broth culture of each bacterium (100 μl) was uniformly spread on the each plate, using sterile cotton swabs. Under aseptic condition, wells (6 mm) were made using sterilized stainless steel cork borer. The *Penicillium* sp. MEAE and negative control (solvent only) was dissolved in 10% aqueous DMSO, obtained different concentrations (50, 75, and 100 μg/ml) and they were loaded on marked wells under aseptic conditions with the help of micropipette, then the plates were incubated at 37°C for 24 h. The antibiotic Chloramphenicol (4 μg/ml) was served as positive control. The growth inhibition zone was measured using a ruler and maintained the values in millimeter (mm) as mean ± SD (Prabhu et al., 2010).

#### Fish Maintenance and Breeding

The fishes were maintained at the temperature of 27 ± 2°C with a 14:10 h of light and dark (photoperiod cycle) in 100 L tanks as per the OECD guidelines as well as the modified protocols of Unai Vicario-Pares et al. (2014). The fish embryos were obtained for spawning the adults *Danio* in the breeding tanks with the sex ratio of 3:5 (3 male:5 female) and the spawning was induced in the morning and after 2 h, embryos were collected and washed using embryo medium. The collected embryos (with help of Pasteur pipettes) and fertilized viable eggs were selected using a stereoscopic microscope (Nikon smz800, Kanagawa, Japan).

#### Acute Toxicity Test of *Penicillium* sp. on Zebra Fish

The acute toxicity test of *Penicillium* sp. on zebrafish was performed by following the modified FET test (OECD, 2006, 2013). Fertilized embryos were treated with different concentrations of mycelia extracts (1.0, 0.5, 0.125 mg/ml, 30, 3.0, and 0.5 μg/ml), prepared from the stock solution (1 mg/L), for 4– 124 hpf. The control fish (with solvents) were also maintained in an embryonic medium with 0.1% DMSO and additional sets of control (without solvent and toxicant) was taken. Then, the embryos were incubated at a temperature of 26.0°C ± 1.0°C with 14:10 (Light:Dark) cycle and monitored at respective time intervals (24, 48, 72, and 96 h). During the treatments, the heartbeat, hatching rate, percentage of survival and body length of fish were noticed using an inverted microscope (Nikon TE2000-U) (Manjunatha et al., 2018). Each test was done three times for obtaining accurate results.

#### Fourier Transformed Infrared Spectroscopy (FTIR)

The dried powdered MEAE of *Penicillium* sp. was subjected to FTIR spectrometer analysis through scanning it in the range 400 to 4000 cm^−1^ at a resolution of 4 cm^−1^. These measurements were performed on a Bruker Optics (Germany-made) Tensor 27 model in the diffuse reflectance mode operated at a resolution of 0.4 cm^−1^ in KBr pellets. In brief, 500 mg of sample was mixed with 300 mg of potassium bromide (KBr) powder and pelletized (3 mm dm). The pellets were analyzed through FTIR spectroscopy measurement (Vivek et al., 2012).

#### Gas Chromatography–Mass Spectroscopy (GC–MS)

The GC–MS spectrum of *Penicillium* sp. MEAE was performed in electron ionization (EI) mode on a GC–MS, PerkinElmer, Turbo mass gold, GC model 680 (Boesch, Huenenberg, Switzerland) system. The details of instrumental working condition and sample loading procedure was done as explained by Ragavendran and Natarajan (2015). The bioactive components of the mycelia extract interpreted based on the obtained mass spectra, which were then compared with the data already available in the National Institute of Standards and Technology (NIST) libraries (Devi and Singh, 2013).

### Statistical Analysis

The LC_50_, LC_90_% confidence limits of upper and lower confidence limits, and chi-square values of mortality data were calculated using Probit analysis and the Statistical Package of Social Sciences IBM 20.0 software (SPSS Inc., Chicago, IL, United States). All the obtained results were considered to be statistically significant, at the larval of *P* < 0.05. Acetylcholinesterase inhibition activity measurements were made in triplicates and the ±SD by Graph Pad Prism 5.0 software program.

## Results

### Isolation of Fungi

Totally 7 different fungi were isolated from the soil samples by serial dilution method and the isolated fungi were purified using a point inoculation technique. The fungi were preliminarily identified by using the microscopic (40×), morphological characters and soil fungal identification key manual (Gilman and Abbott, 1957) and the salient features of all the isolates have been depicted in Table 1. Based on the above results (microscopy and morphological features), the isolated fungi have been identified as: *Penicillium* sp. *A. niger*, *A. flavus*, *A. parasiticus*, *Rhizopus* sp. *Mucor* sp. and *Aspergillus* sp. Then, the *Penicillium* sp. was sub-cultured in the PDA media, for preparation of pure culture and used for further study.

**Table 1:** Macroscopic and microscopic characters of isolated fungi.

S. no	Name of the isolated fungi	Morphological features (on medium)		Microscopic observation	Reference
		**Front view**	**Back view**		
1	*Penicillium* sp.	Fast growth, consists of a width of 05–09 mm (after 07 days), colony shown dark green color and granular powdery.	Colonies were pale yellow in color	Conidiophores are connected to the septate hyphae. The shape of conidia was shown spheraidal to sub-spheroidal. Characteristically fungal thallus showed in microscopic viz. threads or filaments.	Raper and Thom, 1949; Pitt, 1975, 1979, 1988
2	*Aspergillus* sp.	Rapidly grown. The colonies are green, velutinous and uniseriate. Conidial heads develop, within 24/48 h	White to tan to pale yellow in color. Coloration or shade can be dependent on the media	In microscopic observation hyaline, septate observed like tree- or fan-like branching. 2.5/8 mm wide. Stipes may resemble hyphae of zygomycetes.	Balajee et al., 2004
3	*Aspergillus niger*	Colonies shades in black and brown color. Colony diameters were noted as 43–51 mm (after 8 days of incubation at 26°C; PDA). Mycelia mat look like white cream to brown in thick mat of floccose mycelia.	Reverse was brown.	Heads of conidia were ball and biseriate in shape with extensive spherical vesicle (37–52 μm). The stipe measured 430 - 670 × 7 - 13 μm with smooth and slightly brown in color.	Raper and Fennell, 1965; Klich and Pitt, 1988; McClenny, 2005
4	*Aspergillus flavus*	Colony were normally t, velvety slightly covered with regular margins. The difference in color i.e., white color in initially, afterward grayish yellow to blue green; margins are white, radially sulcate; exudates absent.	Pale yellowish in reverse	Conidiophores from substrate and hyphae is colorless, soft, hyaline and thick walled. Head of conidial are diverged. Flask-shaped phialides were observed. Few phialides openly bore on mycelium	Raper and Fennell, 1965; Klich and Pitt, 1986
5	*Aspergillus parasiticus*	Green color colony with white mycelia and unsmooth. It is produced reproduction rings and exudates are lacked.	Dull brown color, conidia on the plate were reduced the thick white mycelia under the colonies.	Conidia heads are uniserite with spread from, which a few were slant at the corner of the stalk on microscopic view.	Samson and Pitt, 1990; Klich, 2002
6	*Rhizopus* sp.	Colonies grew very fast and appeared as white cotton-like colonies then became brownish-gray to blackish-gray depending on the age of sporulation.	Reverse side of the plates was tan.	Rhizoids were found at the junctions of the stolons and sporangiophores, hyphae are broad and chlamydospore, colony look like following features: ribbonlike broad and hyphae are extensive-angle are sub-divided.	Inui et al., 1965; Schipper, 1984
7	*Mucor* sp.	Colonies were grown rapidly at 32°C on PDA was expanding, whitish and hairy.	pale-yellow color in reverse	Broad hyphae which are scarcely or non septate, ellipsoidal, sporangiosphores (4–7 μm diameter) are long, smooth-walled may be branched and terminate in a round spore-filled sporangia (50–300 μm diameter).	Schipper, 1978

### Preliminary Larvicidal Activity

The mycelia extract of the isolated fungi were screened preliminarily and secondary metabolites were tested for larvicidal activity against 1st to 4th instar *Ae. aegypti* and *Cx. quinquefasciatus* larvae. The fungal MEAE of *Penicillium* sp. exhibited superior toxicity against *Ae. aegypti* 3rd instar larvae with LC_50_ value of 6.874 and LC_90_ = 12.879 μg/ml after 24 h treatment, followed by *A. niger* (LC_50_ = 39.293 and LC_90_ = 87.65 μg/ml), *A. flavus* (LC_50_ = 46.381 and LC_90_ = 110.791 μg/ml), *A. parasiticus* (LC_50_= 50.228 and LC_90_ = 147.023 μg/ml), *Rhizopus* sp. (LC_50_= 56.062 and LC_90_ = 210.908 μg/ml), *Mucor* sp. (LC_50_ = 108.363 and LC_90_ = 464.026 μg/ml), *Aspergillus* sp. (LC_50_ = 238.273 and LC_90_ = 962.642 μg/ml). The isolated fungal mycelia metabolites treated larvae exhibited mortality rate of following order: *Aspergillus* sp. > *Mucor* sp. > *Rhizopus* sp. > *A. parasites* > *A. flavus* > *A. niger* > *Penicillum* sp. The *Penicillium* sp. only revealed higher larvicidal activity when compared with the other fungi and hence it was selected for further larvicidal tests.

### Larvicidal Activity of Potential Fungus

The larvicidal effect of fungal MEAE prepared from *Penicillium* sp. has shown excellent activity against 1–4th instar larvae of the *Ae. aegypti* (Table 2) and the larval mortality was noticed after 24 h and the control did not show nil mortality. The metabolite treated 1, 2 and 4th instar larvae (of *Ae. aegypti*) revealed high effect at the maximum concentration (500 μg/ml), and the mortality started within 8 h of exposure. More than 50% larval death occurred within the 12 h. Similar effect was observed against *Cx. quinquefasciatus* at a dose of 500 μg/ml. Similarly, 2nd instar larvae also affected at 300 μg/ml concentration where mortality occurred 100% after 24 h post-treatment. The lowest concentration of metabolite arrested the larval growth and development. Overall, we obtained the *Penicillium* sp. mycelia metabolite was exhibited the results in dose and time dependent death rate.

**Table 2:** Larvicidal activity of MEAE of *Penicillium* sp. against *Ae. aegypti* and *Cx. quinquefasciatus.*

Mosquito species	Larval stages	Concentrations (μg/ml)	LC_50_ (LCL-UCL)	LC_50_ (LCL-UCL)	χ^2^ *df* = 10	*P* value
*Ae. aegypti*	I	100 200 300 500	7.000 (0.582–21.211)	12.541 (1.507–32.455)	2.320	0.993
	II	100 200 300 500	13.943 (2.290–33.467)	23.761 (5.208–49.927)	3.635	0.962
	III	100 200 300 500	18.129 (3.330–41.235)	30.923 (7.586–61.603)	2.766	0.986
	IV	100 200 300 500	25.212 (6.005–51.939)	41.696 (12.797–76.217)	2.040	0.996
*Cx. quinquefasciatus*	I	100 200 300 500	6.554 (0.576–19.726)	11.486 (1.424–29.757)	2.492	0.991
	II	100 200 300 500	5.487 (0.276–19.022)	10.366 (0.821–29.969)	1.948	0.997
	III	100 200 300 500	6.874 (0.435–22.312)	12.879 (1.258–34.786)	4.364	0.929
	IV	100 200 300 500	6.892 (0.254–24.418)	13.865 (0.905–39.819)	5.180	0.879

*Control (deionized water with 10% DMSO) – nil mortality. LC_50,_ LC_90_– lethal concentration that kills 50%, 90% of the exposed larvae, LCL, UCL = lower, upper confidence limit, df – degree of freedom, χ^2^ – chi-square values are significant at P < 0.05 levels. Mean value of triplicates.*

### Neurobehavioral Toxicity

**FIGURE 1:**
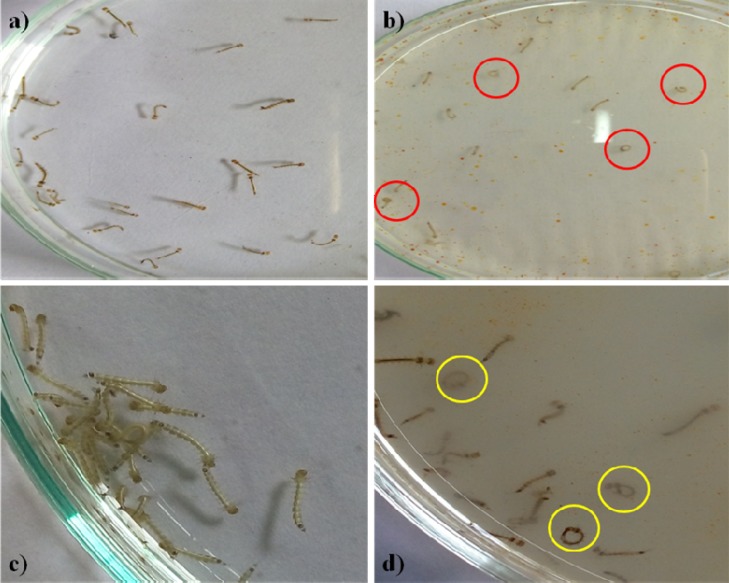
Fourth instar larvae **(a)**. Control *Aedes aegypti* **(b)**. Treated larvae (500 μg/ml) **(c)**. Control *Culex quinquefasciatus* **(d)**. Treated with *Penicilluim* sp. metabolite (500 μg/ml). The red and yellow circle indicates the aggressive anal gill biting behavior.

**FIGURE 2:**
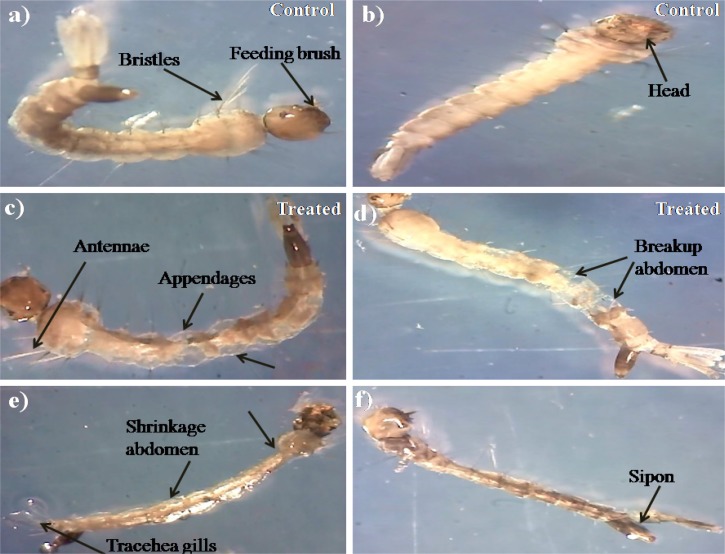
Stereo microscopic view of 4th instar **(a,b)** control larvae of *Ae. aegypti* and *Cx. quinquefasciatus* of midgut, thorax, and anal gill parts. **(c–f)** *Ae. aegypti* and *Cx. quinquefasciatus* treated with *Penicillium* sp. mycelium extract at 500 μg/ml concentration and extract treatment induced toxic effects on many regions of the body (including thorax, abdomen, anal gills, (black arrows) indicates loss of external hairs, crumbled epithelial layer of the outer cuticle and shrinkage of the larvae).

The *Penicillum* sp. mycelia metabolites have exerted neurobehavioral toxicity on 4th instar larvae of the tested mosquitoes (Figure 1a,c). The control larvae have shown normal behaviors and after 30 min exposure of extract, the larvae have exhibited energetic and restless movement with an increased exposure periods i.e., unnatural restlessness and excitation, movements was altered, forceful self bite of anal papillae and mouth parts leading to the development of circle-shaped structures (Figure 1b,d). It was noticed that these continuous behaviors and orientation symptoms of treated larvae and larvae became more irritated and noticed their up and down wriggling movements and several larvae exhibited vibrating movements and paralytic symptoms. Morphological changes were noticed in the treated larvae with damaged anal papillae area and the cuticle layers (Figure 2).

### Acetylcholinesterase Inhibition Test of 4th Instar Larvae

**FIGURE 3:**
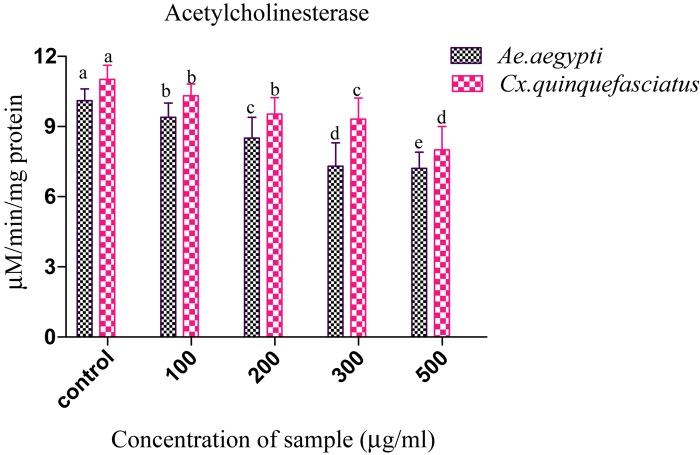
Acetylcholinesterase (AchE) inhibition of *Ae. aegypti* and *Cx. quinquefasciatus* after treatment with *Penicillium* sp. Statistical values followed by the same letter are not significantly differences according to Tukey’s HSD test at *P* < 0.05 (one way ANOVA).

Presently, the *Penicillium* sp. metabolites exposed to 4th instar larvae of *Ae. aegypti* caused reduced AchE level (9.35 ± 0.2 to 7.3 ± 0.7 μM/min/mg larval protein; *F_4_* = 311.93; *P* < 0.05). Similar trend also observed in *Cx. quinquefasciatus* larval (11.01 ± 0.4 to 8.1 ± 0.4 μM/min/mg larval protein; *F_4_* = 186.26; *P* < 0.05) (Figure 3). At the concentration of 100 μg/ml metabolites, the AchE activity was notably inhibited and the maximum inhibition was observed at 500 μg/ml. The untreated larva has shown only enzyme. The enzyme inhibition activity results clearly confirmed the dose dependent approach of metabolites. A *post hoc* Tukey’s HSD test showed significant difference in the inhibition rates based on the concentration of metabolite. A maximum inhibition was observed in *Ae. aegypti* larvae than the *Cx. quinquefasciatus*.

### Histopathological Study

**FIGURE 4:**
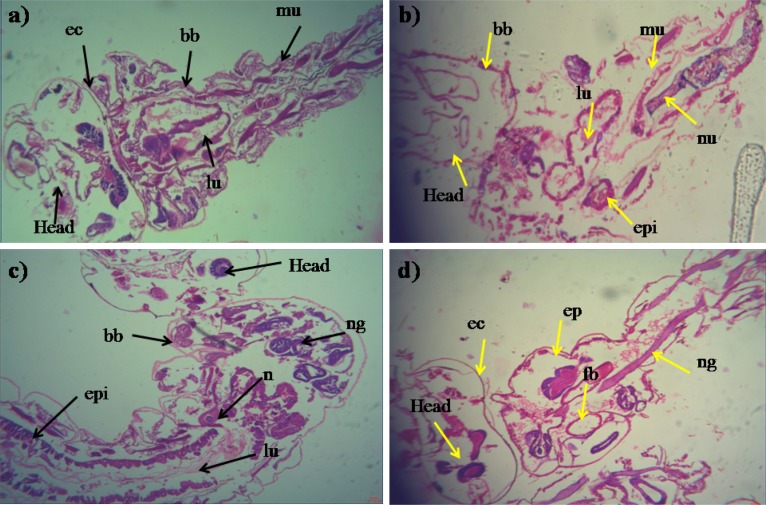
Histopathology profiles of treated 4th instar larvae of *Ae. aegypti* and *Cx. quinquefasciatus*: **(a–c)** Control, **(b–d)** treated with the *Penicillium* sp. MEAE (500 μg/ml), Black arrow represents control larvae structures parts, yellow arrow indicates the treated gut epithelium (epi) and muscles (mu), a nerve ganglion (ng), intact neuropile (nu), damaged gastric (enteric) caeca (gc), brush border (bb), peritrophic matrix (pm) and gut lumen (lu). Magnification is 200×, respectively.

During histopathological study, *Ae. aegypti* treated larvae showed histological alterations in the digestive tract, midgut, cortex with hyperplasia of gut epithelial cells, collapsed brush border, broken membranes and also observed cytoplasmic masses (Figure 4b). On the other hand the, *Cx. quinquefasciatus* larvae showed complete disintegration of abdominal region especially the mid-gut and caeca, and the epithelial layer was disorganized with most of the cells disappeared (Figure 4d). Both the untreated larvae exhibited the midgut epithelium with a single layer of digestive cells exhibiting well developed brush border, cell membrane and cytoplasm regions (Figure 4a–c).

### Antibacterial Activity

Presently, antibacterial activity of *Penicillium* sp. MEAE was tested against 7 Gram-positive and negative bacterial pathogens with 3 different concentrations (50, 75, and 100 μg/ml) of metabolites and the results are presented in Supplementary Figure 1 and Table 3. The maximum inhibitory zone was caused by *Shigella dysenteriae* (31.2 mm), followed by *K. pneumoniae* (31.1 mm) and *S. boydii* (30.2 mm). No zone formation was observed in the DMSO (Negative control). The antibacterial efficacy of fungal is mainly depends on the dose of the mycelia metabolites that are more specific to Gram negative bacteria than Gram-positive organisms.

**Table 3:** Antibacterial activity of MEAE of *Penicillium* sp.

Name of the strains	Zone of inhibition in diameter (mm) Concentration of sample (μg/ml)			
	50	75	100	Chloramphenicol (Positive control)
*S. typhi*	19.0 ± 1.1	25.0 ± 0.3	28.5 ± 1.3	34.0 ± 1.0
*S. aureus*	20.2 ± 0.7	24.3 ± 0.1	29.0 ± 1.0	33.1 ± 0.9
*E. coli*	18.2 ± 1.0	23.3 ± 0.0	25.4 ± 0.5	35.2 ± 0.4
*S. boydii*	25.0 ± 0.5	29.4 ± 1.6	30.2 ± 0.8	38.3 ± 0.2
*K. pneumoniae*	18.2 ± 0.7	25.1 ± 0.4	31.1 ± 0.6	33.0 ± 1.2
*E. faecalis*	20.1 ± 0.6	24.3 ± 0.7	28.0 ± 1.2	30.2 ± 0.5
*S. dysenteriae*	22.3 ± 1.6	24.3 ± 0.9	31.2 ± 1.1	33.1 ± 0.0

*DMSO (Negative control) nil zone of inhibition.*

### Toxicity Assay of Zebrafish

The mycelia extract treated embryos have shown the body length with slight inhibition in the higher concentration of mycelia extract when compared to the untreated groups (Supplementary Figures 2C,D). The hatching out of the zebrafish embryos was noticed as 98.43% after 72 h in the control group. The embryo hatching rate was completely decreased at 1 mg/ml concentration (as compared to control and 0.1% DMSO groups) (Supplementary Figure 2A). While, the embryos exposed to MEAE mycelia extract and the metabolites could not induce any deformities (or) an increase in the pericardial area. But, the heart beat rate was notably reduced in the larvae of all exposure groups than control (Supplementary Figure 2B), The maximum concentration (1 mg/ml) of metabolites exerted slight malformation of embryos pericardial, yolk sac edema and also spine deformation occurred (Supplementary Figures 3A,B).

### FTIR and GC–MS Analysis of *Penicillium* sp. Metabolites

Fourier Transformed Infrared spectrum of the *Penicillium* sp. MEAE revealed the presence of various absorption peaks. The notable peak was observed at 3431.31 cm^−1^ that may be attributed to the O–H stretching of aromatic compounds. The intense peak at 2922.59 cm^−1^ could be assigned to the C–H stretching of alkenes. The medium peak at 1631.41 cm^−1^ could be attributed to the N–H bending of the primary amines. The peak at 1383.68 cm^−1^ could be corresponds to the C–H bending of alkyls, respectively. The peak at 1323.89 cm^−1^ region of spectrum corresponds to the C–N stretching arising from strong aromatic amine groups. A medium peak at 1105.01 cm^−1^ corresponds to C–N stretching aliphatic amines. The absorption band appearing in 1028.84 cm^−1^ denote the presence of bending = C–H vibrations due to carboxylic acids. The obtained bands at 872.63 and 619.03 cm^−1^ corresponds to the N–H wagging and C = C–H bending of primary amines and alkyl halides, respectively (Supplementary Figure 4 and Table 4). The GC–MS analysis of the *Penicillium* sp. MEAE showed several compounds, based on the peak values and with the aid of NIST library. The identified compounds structure, molecular formula and molecular weight of the identified compounds are presented in Supplementary Figure 5 and Table 5.

**Table 4:** Identification of functional groups from MEAE of *Penicillium* sp. by FT-IR analysis.

S. no.	Wave numbers cm^−1^	Peak assignment	Mode of vibration	Functional groups
1	3431.71	O–H stretching	Medium	Phenols
2	2922.59	C–H stretching	Medium	Alkenes
3	1631.48	N–H bending	Medium	Primary amine
4	1383.68	CH3 C–H bending	Medium	Alkyls
5	1323.89	C–N stretching	Strong	Aromatic amines
6	1105.01	C–N stretching	Medium	Aliphatic amines
7	1028.84	=C–H bending	Medium	Carboxylic acids
8	872.63	N–H wagging	Strong, broad	Primary or Secondary amines
9	619.03	–C≡C–H bending	Broad	Alkyl halides

**Table 5:** GCMS analysis of MEAE of *Penicillium* sp.

S. no.	Name of the compound	Molecular formula	Molecular weight	Biological activity	Reference
1.	Indene	C_9_H_8_	116.16	Fungicidal, antimicrobial and anticoagulative activity	Song et al., 2008
2.	1-Undecanol	C_11_H_24_O	172.31	Antibacterial activity	Selvamangai and Bhaskar, 2012
3.	7-Chloro-4-(phynylazo) quinoline	C_15_H_10_C_1_N_3_	267.71	Antibacterial and Antimalarial	Rudrapal and Chetia, 2010; Nqoro et al., 2017
4.	Cyclopentasiloxane, decamethyl	C_10_H_30_O_5_SI_5_	370.77	Antimicrobial activity	Jasim et al., 2015
5.	3-(Methylthio)pent-4-yn-1-O1	C_6_H_10_S	114.20	Not known activity	.......
6.	1-Decene	C_10_H_20_	140.27	Not known activity	Siddiquee et al., 2012
7.	Boron trichloride	BCl_3_	117.17	Anti-malarial and Anti-inflammatory activity	Marella et al., 2013
8.	3-Tetradecene, (E)-	C_14_H_28_	196.37	Antimicrobial activity	Paritala et al., 2015
9.	Cycloheptasiloxane, tetra decamethyl	C_14_H_42_O_7_Si_7_	519.07	Antifungal activity	Moustafa et al., 2013
10.	Cetene	C_1_OH_20_	140.27	Antimicrobial activity	Bouaziz et al., 2017
11.	1-Octadecene	C_18_H_36_	252.48	Larvicidal activity	Sina et al., 2015; Jayaseelan et al., 2018
12.	1-Nonadecene	C_19_H_38_	266.51	Larvicidal activity	Rawani et al., 2017
13.	Pthalic acid	C_8_H_6_O_4_	166.14	Antimicrobial activity	Piscopo et al., 1984
14.	Cyclononasiloxane, octa Decamethyl	C_18_H_54_O_9_Si_9_	667.38	Antioxidant and Antimicrobial	Mushtaq et al., 2013
15.	Dibutyl phthalate	C_16_H_22_O_4_	278.34	Antiviral and Antitumor activity	Osama et al., 2015
16.	1-Octacosanol	C_28_H_58_O	410.77	Actinociceptive and antiinflammatory activity	Piatak and Reimann, 1970
17.	6-Octadecynoic acid	C_18_H_34_O	282.46	Antimicrobial activity	Sanabria-Ríos et al., 2014
18.	Octadecanethioic acid	C_18_H_36_OS	300.54	Not known activity	........
19.	9-Octadecenoic acid	C_18_H_34_O_2_	282.46	Larvicidal and Antibacterial activity	Hemalatha et al., 2015; Silva et al., 2015; Arora et al., 2017
20.	1,2 Benzenedicarboxylic acid, bis (2-methylpropyl)ester	C_16_H_22_O_4_	278.34	Antimicrobial activity	Nakalembe and Kabasa, 2012
21.	Cyclobutane	C_12_H_22_	56.10	Larvicidal activity	Anjana and Thoppil, 2013
22.	Bendazol	C_14_H_12_N_2_	208.26	......	.......
23.	Carbonic acid	C_12_H_22_	62.02	......	.......

## Discussion

### Isolation of Fungi

Fungi are highly complex group having variety of species with diverse metabolites that could kill mosquitoes. Fungi and actinomycetes are the greatest source for the production of diverse secondary metabolites (Vijayan and Balaraman, 1991). They act as a better source of powerful agents, which could be used toward the bio-control of pests and parasites (Rose, 1979). Generally, the number of fungi present in natural sources are very high when compared to the other microbes. They are being isolated from various sources by using different methods. Most of the fungal species are identified based on their morphological appearance (Rekha et al., 2015). Presently, seven different fungal species were isolated from soil viz, *Penicillium* sp. *A. niger*, *A. flavus*, *A. parasiticus*, *Rhizopus* sp. *Mucor* sp. and *Aspergillus* sp. Earlier, Kostadinova et al. (2009) have reported that the isolation and identification of fungi from the soils and their most frequently obtained forms belonged to the genera; *Penicillium*, *Aspergillus*, *Cladosporium*, *Alternaria*, *Geomyces*, and *Lecanicillium*. Manivannan and Kathiresan (2007) have reported the isolation of *P. fellutanum* from the rhizosphere the regions. Interestingly, the present findings are in agreement the results of Kumari et al. (2005) who have isolated 46 fungi (including *Aspergillus* and *Penicillium*) from 40 different soil samples (Gugnani and Shrivastav, 1972; Mini et al., 2012).

### Larvicidal Activity

Microbe based control agents offer an alternative to chemical pest/insect control, because it can be more selective than chemical insecticides (Abdel-Baky and Abdel-Salam, 2003). The different types of biolarvicidal agents have been produced by several researchers with effective LC_50_ and LC_90_ values against many diseases causing mosquito-borne vectors (Kamaraj et al., 2009). The fungus metabolite has created highest mortalities in mosquito populations particularly in *Culex* sp. (Axtell et al., 1982) and *Anopheles* sp. (Kerwin and Washino, 1986). McCray et al. (1973) have proved that the indigenous soil fungus might successfully infect and kill the larvae of test mosquitoes with 100% mortality. Recently, several researchers have focused on identifying more potential mosquitocidal agents from biological resources toward the control of disease-transmitting mosquito vectors. Presently, the mycelial extracts of 7 different soil fungi were tested for larvicidal activity against the *Ae. aegypti* and *Cx. quinquefasciatus* larvae. The MEAE of *Penicillium* sp. was found to be more toxic than the other fungi. Presently, *Penicillium* sp. was found be more effective on all larval stages of *Cx. quinquefasciatus*. Presently, high LC_50_ and LC_90_ values against *Ae. aegypti* was found in least doses of metabolites when compared to the earlier report on *Penicillium daleae* mycelium extract (Ragavendran et al., 2017b). Earlier, Ragavendran et al. (2017a) reported that the *Beauveria bassiana* MEAE exerted larvicidal effect on 1st to 4th instar larvae of tested mosquitoes. Also, Govindarajan et al. (2005) have reported the mycelial extracts from various fungi i.e., *Aspergillus flavus*, *A. parasiticus*, *Penicillium falcicum*, *Fusarium vasinfectum* and *Trichoderma viride* have more toxicity caused against *Cx. quinquefaciatus*. Earlier, Cooper and Sweeney (1986) and Seif and Shaarawi (2003) reported that the *Tolypocladium cylindrosporum* and *Culicinomyces clavisporus* have shown toxic effect on larvae of selected mosquitoes. Further, Thomas and Read (2007) also investigated the fungal derived products that were found to be toxic against the tested mosquitoes and they found only lower toxicity against brine shrimp organisms. Balaraman et al. (1979) investigated the larval bio-effect of *Metarhizium anisopliae*, *Beauveria tenella* and *Fusarium oxysporum*. The outcome of present investigation, found that high potential utility of fungal metabolites acted as a complement for the existing larval control measures. This approach is being supported by Singh and Prakash (2012) who have demonstrated that the fungal metabolites could serve as a new strategy for filariasis and dengue control. The metabolites of *L. giganteum* showed a significant effect against *Cx. quinquefasciatus* and *Ae. aegypti* (Scholte et al., 2005).

### Behavioral Studies

Shaalan et al. (2005) reported that secondary metabolites from fungus had shown the growth inhibitory effects on the various developmental stages of different mosquitoes i.e., delays in larval development, extended pupal durations, molting inhibition, morphological abnormalities, and mortality rate especially in molting and melanization processes. Presently, it was noticed that the *Penicillium* sp. MEAE-treated larvae displayed an unusual movement, excitation, horizontal and vertical aggressive movements. Similar kind of result was earlier obtained by Choochote et al. (2005). Chaithong et al. (2006) have reported the *Pepper longum* ethanolic extract treated *Ae. aegypti* larvae exhibited morphological alterations such as anal papillae shrinkage and cuticle dysfunction. Earlier, Kumar et al. (2010) who have studied the peppercorns extract treated *Aedes* sp. larvae that revealed deformation, vigorous self anal biting and shrinkage of external membranes. Earlier, Becker et al. (2010) studied the role of anal papillae in electrolyte balance regulation, which is necessary for the development of the life functions. Perumalsamy et al. (2010) have proved that the structural malformation of anal papillae may be intrinsically connected with the death of the treated larvae.

### Acetylcholinesterase Assay

The organophosphorus and carbamate are the major cluster which served as target to the acetylcholinesterase (AChE) involved in the synaptic communication of nerve impulse in living beings (Yu et al., 2001). Microbial/plant secondary metabolites have been reported to decreases the AchE activity. The dose dependent response of α-carboxylesterase activity was noticed in the larvae of *Choristoneura rosaceana*, exposed to neem oil (Smirle et al., 1996). Likewise, Ortego et al. (1999) reported Limnoid has the potential as larvicide (from *Scutellaria alpine*) and found *Leptinotarsa decemlineata* esterase activity was significantly reduced in the coleopteran larvae. The present study showed that *Penicillium* sp. metabolite inhibits the AchE enzyme in the dengue and filarial vectors. The inhibition of AChE enzymes by mycelia extract was dose dependent and direct connections with the insect toxicity and which leads to eventual death of the organism. Likewise, Jukic et al. (2007) and Lopez-Hernandez et al. (2009) have studied the inhibitory property of AChE activity of monoterpenoids against various pests.

### Histopathological Profiles of 4th Instar Larvae

The histopathological analysis of the treated larvae exhibited several alterations namely shrinkage of abdominal region, and damages to the midgut, muscles, and epithelial layers. Abinaya et al. (2018) have studied the *Bacillus licheniformis* exopolysaccharide treatment on 4th instar larvae of *An. stephensi* and *Ae. aegypti* and found different histological changes such as the gastric caeca, muscles shrinkage, damaged and disorganized nerve cord ganglia. Earlier, Farida et al. (2018) studied the *B. bassiana* metabolites toxicity on 4th instar larvae of *Cx. pipiens* and observed many histological changes and malformation in the treated larval body and tissues, especially in the adipose cells, cuticles and midgut. And, Ragavendran et al. (2017b) have reported that the midgut cells of tested mosquitoes (4th-instar larvae) had swelling in the gut lumen, reduced intercellular contents and degeneration of nuclei, after treated with *P. daleae* mycelium extract.

### Antibacterial Activity

Natural products play an essential role in the discovery and development of drugs for the treatment of human diseases and microbial environment is an important source of novel bioactive agents (Newman et al., 2003). Since, the discovery of penicillin, the micromycetes have been famous as producers of antibiotics and other secondary metabolites with biological activity (Rancic et al., 2006). *Penicillium* sp have been proved as being abundant producers of many bioactive secondary metabolites/mycotoxins (Nicoletti et al., 2014) like citrinin and penicillic acid has shown potent antimicrobial activity (Kang et al., 2007; Yu et al., 2010). Presently, *Penicillium* sp. mycelia extract was screened for antibacterial activity against human pathogens. The mycelia metabolites exhibited significant antibacterial activity against all tested pathogenic bacteria particularly, *K. pneumonia*e and *Salmonella dysentriae* had maximum antibacterial activity. Gram-negative bacteria possess an outer membrane and a unique periplasmic space (Duffy and Power, 2001) whereas the Gram-positive bacteria are found to be more susceptible because they have only an outer peptidoglycan layer which is not an efficient permeability barrier (Scherrer and Gerhardt, 1971). The increased permeability of the membrane by the insertion of metabolites or free fatty acids can allow internal contents to leak from the cell, which can cause growth inhibition or even death (Wang and Johnson, 1992; Boyaval et al., 1995; Shin et al., 2007). Moreover, Devi et al. (2012) found a maximum antibacterial activity against *Vibrio cholerae*, *E. coli* and *S. aureus* by *Penicillium chrysogenum* bioactive metabolites. Kaur et al. (2014) isolated antimicrobial compound 6-[1,2-dimethyl-6-(2-methyl-allyloxy)-hexyl]-3- (2-methoxy-phenyl)-chromen-4-one from *Penicillium* sp that showed effective growth inhibition against *S. aureus*, *E. coli*, *S. epidermidis* and *Salmonella typhimurium.* Interestingly, Zhuo et al. (2012) reported the *Penicillium commune* bioassay guided fractionated compound xanthocillin X that showed significant antibacterial activity against *S. aureus* and *E. coli*. Specian et al. (2012) isolated the *Diaporthe helianthi* strain and its metabolites was tested against several human pathogenic bacteria. Silva et al. (2011) reported that the secondary metabolites of *A. niger, Curvularia pallescens, Guignardia bidwelii, Paecilomyces variotii* and *Mycelia sterilia* showed antibacterial activity against *S. aureus, B. subtilis, E. faecalis*, *M. luteus, E. coli* and *Pseudomonas aeruginosa*. Awad et al. (2018) have reported that the soil derived fungus, *Trichoderma viridae* mycelia metabolites exhibited significant antibacterial effects on *Bacillus subtilis*, *E. coli* and *Pseudomonas fluorescens*. Danagoudar et al. (2017) also studied the ethyl acetate extract of *Penicillium citrinum* that was tested against the human pathogenic bacteria and *Cx. quinquefasciatus* larvae.

### Zebrafish Toxicity Assay

*Danio rerio*, zebrafish is an important *in vitro* aquatic model test organism being widely used in eco-toxicology fields. The acute aquatic toxicity tests are required for the assessment of biocides and plant based products (Seiler et al., 2014). Presently, the zebra fish embryos body length, hatching rates; heart beat count and survival percentage of *D. rerio* was significantly decreased. Rajaofera et al. (2017) isolated *Bacillus atrophaeus* substances that are non-toxic against *D. rerio* embryo. Further, Cao et al. (2016) have also reported the morphological and physiological changes in the cyhalofop-butyl treated zebrafish, that showed reduced hatching rate of embryos, delayed spontaneous movement, decreased heartbeat, and the reduced body length of larvae (at higher concentrations). On the other hand, Abutaha et al. (2015) isolated the *Cochliobolus spicifer* endophytic fungal extract that was tested for larvicidal as well as zebrafish embryo toxicity but its metabolites did not induce any toxicity symptoms on embryo.

### FTIR Analysis

Presently, the FTIR spectrum of the mycelia extract of *Penicillium* sp. reflects the presence of various functional groups such as phenolic, alkenes, carboxylic acids and amine groups. Devi and Singh (2013) have studied the FT-IR spectral characteristics of *Colletotrichum gloeosporioides* fungus that revealed the functional groups of phenolic group, carboxylic acids (C–H group), aliphatic amines (C–N stretch), alkenes (C–H stretch), primary or secondary amines, and carboxy groups (C–C, stretch). Earlier, Ragavendran and Natarajan (2015) also reported the active functional group from FTIR spectrum of *Aspergillus terreus* mycelia extract at the peak value of 1023.59 cm^−1^ corresponding to C–O and C = N, which indicated the presence of carboxylic acid and amine groups. In addition to this, Abutaha et al. (2015) reported the *C. spicifer* fungi extract that revealed different functional groups such as carbonyl, methyl, phenol, amide and amines, respectively.

### GCMS Analysis

The GC–MS analysis of the *Penicillium* sp. mycelial extract showed the presence of 1-Octadecene (5.10%) and 9-Octadecenoic acid (12.33%) which may responsible for mosquitocidal properties. Recently, Jeyasankar and Chinnamani (2018) reported that the *Solonum pseudocapsicum* extract revealed many compounds viz; n-Hexadecanoic acid, 9-Hexadecenoic acid and 9,12-Octadecadienoic acid (Z,Z)-, methyl ester that were responsible for larvicidal and pupicidal properties. Senthilkumar et al. (2014) have reported the insecticidal activity of *Phomopsis* sp because of the presence of Dodecanoic acid ethylester and Phthalic acid, octyl 2-pentyl ester with better insecticidal property. The main chemical constituents were identified by using GC–MS viz: Phenol, 2, 4-bis (1,1 dimethylethyl), 1-Hexadecene, 1-Hexadecanol, Hexadecanoic acid, octadecanoic acid methyl ester and 1-Nonadecene (Devi and Singh, 2013). On the other hand, Stanly Pradeep et al. (2015) have reported the soil fungus *Fusarium moniliforme* derived compounds (2-(4-((3E, 5E)-14-aminotetradeca-3,5-dienyloxy)butyl)-1,2,3,4-tetrahydroisoquinolin-4-ol) that exhibited potent mosquito larvicidal activity against 3–4th instar larvae of *Ae. aegypti* and *An. stephensi*, respectively. Ealier, Elsa Lycias Joel and Valentin Bhimba (2013) through GCMS analysis of marine fungus *Meyerozyma guilliermondii* found mainly secondary metabolites viz*:* Isohexyl neopentyl ester, Tridecane, Pentadecane, Isobutyl undecyl ester, 1-Nonadecanol, and 9-Octadecenoic acid. Lorenzen et al. (1996) reported the isolation of 11-hydroxy-4-methyl-2,4,6-dodecatrienoic acid from *Mucor* sp. which showed nematocidal activity.

## Conclusion

In conclusion, totally seven indigenous fungal species (*Penicillium* sp. *A. niger*, *A. flavus*, *A. parasiticus*, *Rhizopus* sp. *Mucor* sp. and *Aspergillus* sp.) were isolated from soils. Among them, only the *Penicillium* sp. showed the better larvicidal, histopathological and antibacterial potential. The fungal MEAE of *Penicillium* sp. expressed good toxicity against *Ae. aegypti* 3rd instar larvae with LC_50_ value of 6.874 and LC_90_ 12.879 μg/ml, after 24 h treatment. After 30 min exposure of metabolites, the behavioral symptoms of treated larvae showed excitation, up and down, horizontal and vertical movements with violent self anal biting features. Morphological changes were observed in the treated larvae as noticed in damaged anal papillae area and the cuticle layers. The *Ae. aegypti* and *Cx. quinquefasciatus* 4th instar larval mortality was directly linked to the inhibition of acetylcholinesterase activities. Histological study of metabolites showed in the digestive tract, midgut, cortex, hyperplasia of gut epithelial cells, brush border collapsed broken membranes and also observed cytoplasm masses. The antibacterial activities of three different concentrations of mycelia metabolites (50, 75, and 100 μL) with seven microorganisms were tested. The maximum zone of growth inhibition was recorded in *S. boydii* (30.2 mm), *S. dysenteriae* (31.2 mm) and *K. pneumoniae* (31.1 mm). Furthermore, the toxicity assay of zebrafish embryo was done using the *Penicillium* sp. mycelia extract after 124 hpf hatched embryo. The treated embryos showed slightly decreased the body length at higher concentration of mycelia extract as compared to untreated groups. The hatching out of the zebrafish embryos was noticed as 98.43% after 72 h in the control group. FTIR spectrum of the mycelia extract of *Penicillium* sp. revealed notable peak at 3431.31 cm^−1^ that could be attributed to the O–H stretching of aromatic compounds. GCMS analysis revealed that the fungus *Penicillium* sp. produced more number of bioactive compounds against tested larvae. The outcome of the results opens a new avenue for *Penicillium* sp. based novel metabolites that could form potential bio-insecticidal agents to control the vector-borne diseases in future.

## Author Contributions

CR and DN designed the research plan and drafted the manuscript. CR, CK, JP, and VM performed the experimental works and data compilation. CR, CK, PP, and GB coordinated the work and discussed the results. CR, VM, and JP performed embryo toxicity test and data compilation. All authors read and approved the final manuscript.

## Conflict of Interest Statement

The authors declare that the research was conducted in the absence of any commercial or financial relationships that could be construed as a potential conflict of interest.
