# Supplementary material for: Larvicidal, Histopathological, Antibacterial Activity of Indigenous Fungus Penicillium sp. Against Aedes aegypti L and Culex quinquefasciatus (Say) (Diptera: Culicidae) and Its Acetylcholinesterase Inhibition and Toxicity Assessment of Zebrafish (Danio rerio)
Source: Front Microbiol. 2019 Mar 18;10:427. doi: 10.3389/fmicb.2019.00427 (PMC6431641; doi:10.3389/fmicb.2019.00427)
Supplement: Supplementary file 1 [file Data_Sheet_1.doc]

**Supplementary materials**

**S-Figure 1** Antibacterial activity of MEAE of *Penicillium* sp.

**S-Figure 2** Toxicity assay of *Penicillium* sp. ME on larvae of zebrafish. **A**) Body length after treatment of larvae. **B**) Hatch rate was evaluated at 72 hpf (n =50). (**C**) Heart beats per minute (bpm) (n = 15). (**D**) Percentage of survival rate of larvae was assessed from 0 to 120 hpf (n =124-240). All data are presented as means ± SD.

**S-Figure 3** Toxicity assay of *Penicillium* sp. metabolites against Zebra fish embryo development **A**). Control (0.1% DMSO treated embryos). **B**). The Zebrafish embryos exposed to 1.0, 0.5, 0.125mg/ml, 30, 3.0, and 0.5 μg/ml of ME. Red arrow indicates cardiac region.

**S-Figure 4** Identification of functional groups from *Penicillium* sp. MEAE.

**S-Figure 5** GCMS analysis of MEAE of *Penicillium* sp*.*

**
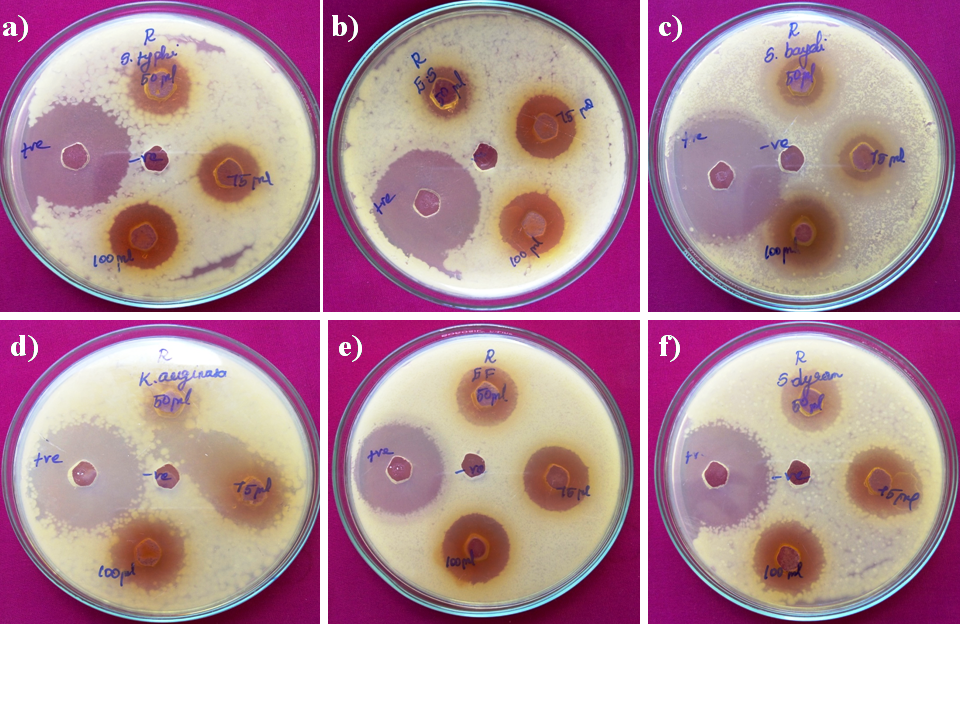
**

**
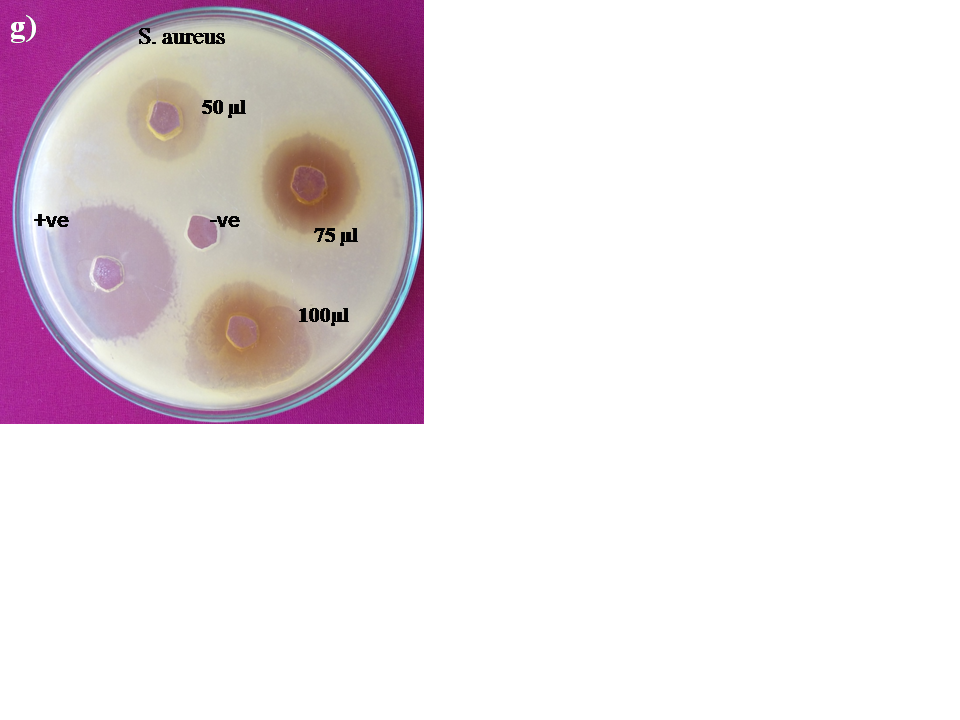
**

**S-Figure 1**

**
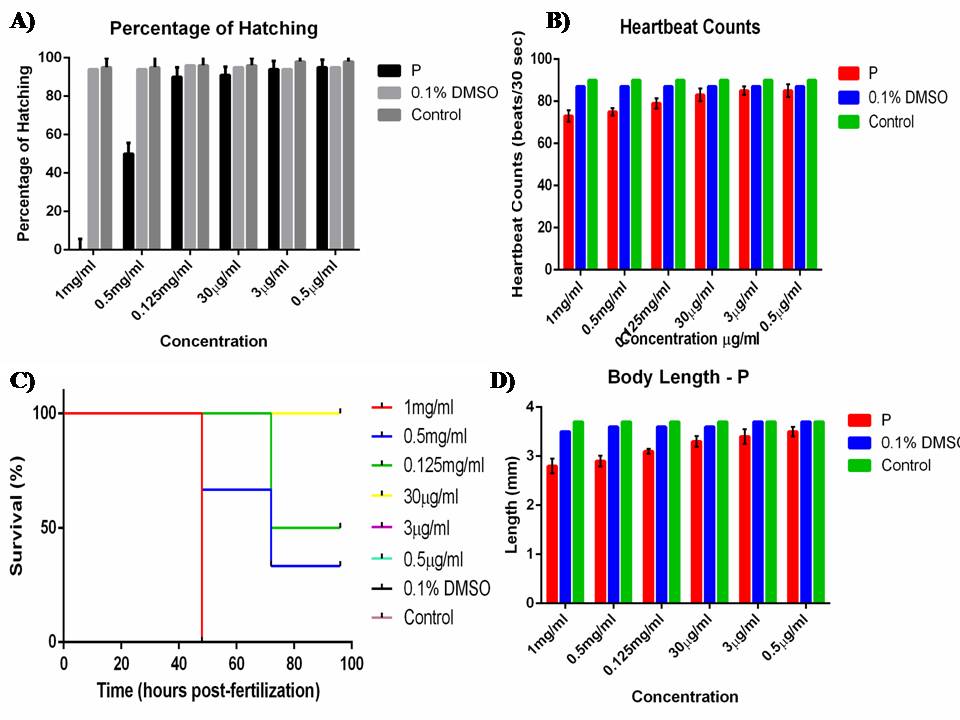
**

**S-Figure 2**

**
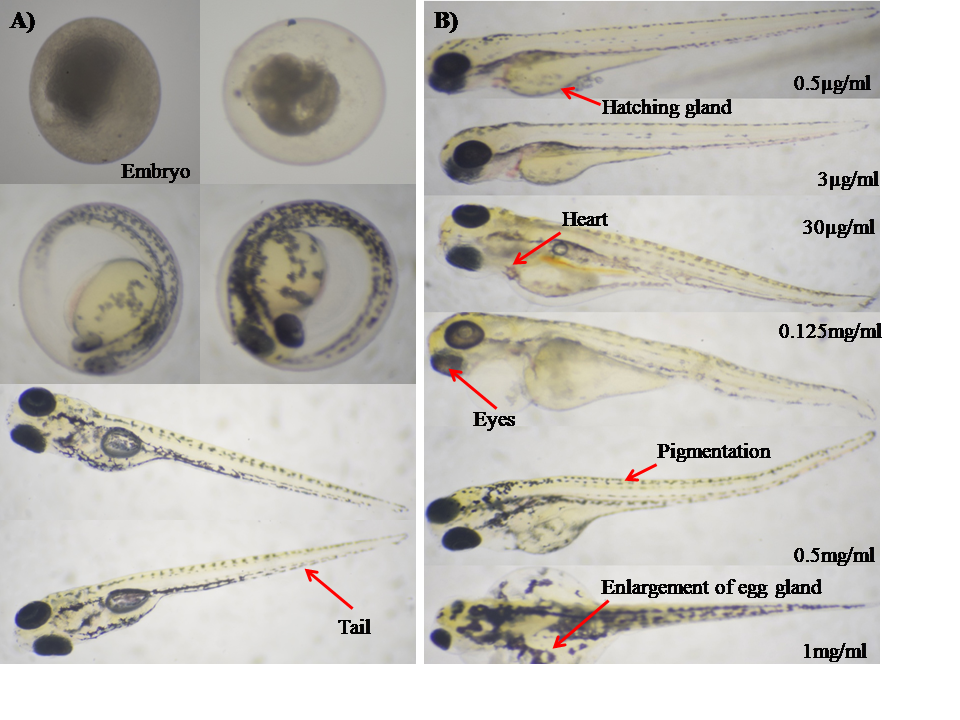
**

**S-Figure 3**


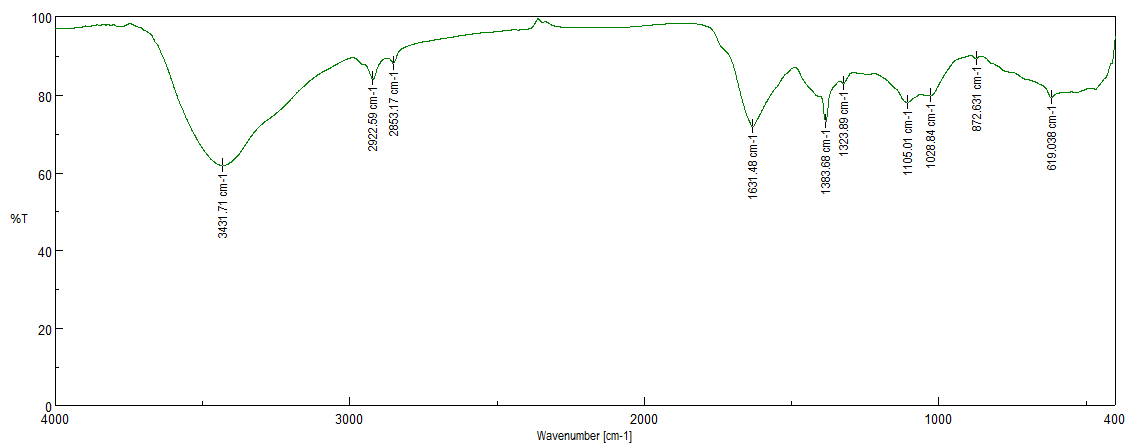


**S-Figure 4**

**S-Figure 5**
